# Systemic Lupus Erythematosus in Primary Care: An Update and Practical Messages for the General Practitioner

**DOI:** 10.3389/fmed.2018.00161

**Published:** 2018-05-29

**Authors:** Irini Gergianaki, George Bertsias

**Affiliations:** Rheumatology, Clinical Immunology and Allergy, University of Crete Faculty of Medicine, Iraklio, Greece

**Keywords:** autoimmune disease, epidemiology, diagnosis, primary care, community, comorbidities

## Abstract

Systemic Lupus Erythematosus (SLE) is a complex chronic autoimmune disease that manifests a wide range of organ involvement. Traditionally, the diagnosis and management of SLE is provided at secondary and tertiary centers to ensure prompt initiation of treatment, adequate control of flares and prevention of irreversible organ damage. Notwithstanding, the role of primary care in SLE is also emerging as there are still significant unmet needs such as the diagnostic delay at the community level and the high burden of therapy- and disease-related comorbidities. In the present review, we summarize practical messages for primary care physicians and general practitioners (GPs) concerning early diagnosis and proper referral of patients with SLE. In addition, we discuss the main comorbidities complicating the disease course and the recommended preventative measures, and we also provide an update on the role and current educational needs of GPs regarding the disease.

## Introduction

Systemic lupus erythematosus (SLE) is a complex autoimmune disease with chronic relapsing-remitting course and variable manifestations leading a spectrum from mild mucocutaneous to devastating, life-threatening illness (1, 2). The clinical profile of lupus is often challenging as the disease can be unpredictable, affecting various organs with variable degree of severity, and is complicated by accrual of organ damage and comorbidities. Traditionally, the diagnosis and management of SLE is provided at secondary and tertiary centers with experience in the disease, to ensure prompt initiation of treatment, early recognition and control of flare-ups, and optimization of medical care during the disease course (3).

Notwithstanding, the role of primary care in routine management of patients with SLE is also emerging. Thus, lupus is no longer considered to be a rare disease and at the community level, there is likely a considerable number of patients who remain undiagnosed or experience significant diagnostic delays (4). Further, the burden of SLE is increasing in a fragmented health care system (5, 6). To optimize patient referral and management, the American College of Rheumatology (ACR) developed relevant guidelines in 1999 (1), which recommend that general practitioners (GPs) should monitor patients with mild stable lupus and manage more severe forms of disease in close collaboration with a specialist. Despite progress, many GPs are concerned about not having sufficient knowledge or experience to tackle SLE (7) and thus, tend to overestimate the consequences that the disease can have for their patients (8). To this end, Lam et al. (2) have published a comprehensive review on lupus for primary care professionals.

The present review aims to provide a contemporary view of SLE at the population level, followed by practical messages to primary care physicians/GPs concerning the early diagnosis and proper referral to rheumatologists. We discuss the main comorbidities complicating the disease course and the recommended preventive measures. We conclude with an update on the role and current educational needs of primary care doctors regarding lupus.

## The changing landscape of SLE

### What causes lupus?

According to the current paradigm, SLE may be triggered in a genetically-susceptible individual by exposure to certain environmental risk factors. Epigenetic modifications (9) mediate the effect of the environment on immunologic responses (10), eventually leading to an inflammatory, autoimmune, multi-systemic disease characterized by autoantibody production and tissue injury (11).

Epidemiologic evidence suggests that increased risk of SLE is associated with exposure to crystalline silica, cigarette smoking, use of oral contraceptives and hormone replacement therapy, while there is an inverse association with alcohol intake (12). Less confirmed associations have been reported for exposure to solvents, pesticides, heavy metals and air pollutants, whereas data regarding other environmental factors or triggers such as ultraviolet light, infections, vaccinations, solvents, pesticides, mercury, obesity, and perinatal factors, are yet inconclusive (12).

### Is lupus a hereditable disease?

A recent study showed that lupus heritability (defined as the proportion of the phenotypic variance explained by genetic factors) is approximately 44%, which is lower than previously reported estimates (up to 66%) (13). The remaining risk may be driven by shared (“familial”) (26%) and non-shared environmental factors (30%) (14, 15).

### Trends in SLE frequency

SLE has long been perceived as a rare disease but most recent studies have disputed this tenet (16). Although reports on SLE occurrence are conflicting, the overall worldwide trend is increasing (17) in terms of both prevalence (5, 18) and incidence (19). An almost 3-fold increase in new cases that was reported during the previous decades (50's−90's) was primarily attributed to better recognition of milder forms of the disease (19). SLE is more common in urban as compared to rural areas and there is circumstantial evidence that lifestyle and environmental factors may account for this difference (20, 21). In view of the significant variations across regions, ethnicities and races, it is of great importance for a GP to be informed about the epidemiology and burden of SLE in her/his population of interest.

### The role of ethnicity

Numerous studies report increased frequency of SLE in non-white people (5- to 9-fold increased incidence and 2- to 3-fold increased prevalence as compared to whites) and in certain ethnicities such as African-Caribbean and South/East Asians (22–28). Further, ethnicities such as Hispanics tend to present with more severe disease, higher activity and organ damage accumulation (19, 27, 29–31), as well as with more comorbidities (increased risk of cardiovascular events). Illustratively, the prevalence of lupus nephritis, one of the most severe disease manifestations, ranges from 20 to 30% of SLE patients in Europe (32) and in the US (33), to more than 60% in certain ethnicities such as Asians (34, 35).

### Gender differences: SLE in men is emerging

A recent review by Rees et al. (5) confirms that females have much higher incidence than males. On average, SLE exhibits a female-to-male ratio ranging from 10–15:1 in adults to 3–5:1 in children (36). Notably, the time of disease onset, clinical manifestations, comorbidities and disease course differ considerably between male and female patients (37). For instance, male patients often have more abrupt onset (38) and manifest more severe disease due to nephritis and serositis, although these findings have not been confirmed by all studies (39, 40). Together, evidence suggests that males may comprise a subgroup of patients with distinct characteristics (41), and that SLE in males is on the rise (20).

### The role of age: SLE appears not only in young adults

SLE can develop at any age (40) and tends to start later in men (typically, 5th to 7th decade of life) than in women (3rd to 7th decade) (5). In many studies, the mean age of SLE onset ranges 35–45 years old, especially at community settings. Data from UK primary care using the Clinical Practice Research Datalink showed that the mean age of SLE diagnosis approximated 49 years in males and 48 years in females; accordingly, GPs should suspect SLE not only in women of younger ages and likewise, they should not disregard the possibility of SLE in people aged 50 or 60 years (42).

Childhood lupus refers to 10–20% of all SLE cases (43) and compared to adults, children present with more renal (odds ratio [OR] 1.55) and neurological (OR 1.64) involvement (44–48). Similar to adults, Caucasian children tend to present with less severe disease as compared to other ethnicities (49–51). Late-onset lupus refers to cases with disease onset after the age of 50. In this group, SLE has more insidious presentations with less specific symptoms which might be a reason for being undiagnosed (52). Thus, late-onset lupus manifests less nephritis and less disease activity (53–55). Unfortunately, the outcome is poorer with increased mortality probably due to comorbid situations and frailty (40).

## When to suspect lupus in primary care?

SLE can affect any organ including the musculoskeletal, skin, hematologic, renal, neuropsychiatric, cardiovascular, and respiratory system. The following points could be helpful in understanding the disease evolution in real-world setting:

First, not all manifestations appear simultaneously and occasionally, a time interval of several months or years may exist between them. In most patients, constitutional (especially fatigue), mucocutaneous and musculoskeletal represent the earliest complaints (56). Thus, data from the UK primary care using the Clinical Practice Research Datalink showed that musculoskeletal symptoms were most frequently (58.6%) recorded in the 5 year-period before SLE definitive diagnosis (42). Conversely, only few patients reported signs of active involvement of the kidneys (proteinuria or cellular casts) or other major organs (serositis, seizures, or psychosis) prior to SLE diagnosis (42). It should be noted, however, that no specific pattern of symptoms/organ involvement combinations exists in SLE and overall, a milder disease pattern may prevail at the community level as opposed to large referral centers (20). These data imply that GPs are most likely to suspect SLE in patients who present with milder symptoms from the skin and joints, and practically, since arthritis/arthralgia are the most common initial symptoms of the disease, every young woman with these symptoms should be evaluated for possible SLE (57). Notwithstanding, major organ disease such as nephritis can sometimes be the presenting manifestation of lupus, thus emphasizing the role of primary care physicians in identifying early signs of renal involvement with simple, inexpensive tools (e.g., urinalysis). This is particularly important considering the 2012 Systemic Lupus International Cooperating Clinics (SLICC) criteria which enable the classification of SLE patients based solely on biopsy-proven lupus nephritis and positive autoantibodies (58). Importantly, early effective immunosuppressive therapy can improve renal outcomes in such patients (59).

Next, increased healthcare utilization during the time preceding SLE diagnosis has been reported. The median number of GP consultations increased during the 5-year interval preceding SLE diagnosis, i.e., from median 1 in the 48–54 months before diagnosis to 38 in the 0–12 months before diagnosis (42). Interestingly, a study performed in 682 children and young patients (aged 10–24 years) with SLE confirmed that they had significantly more health care visits than controls in the year before diagnosis with most (39%) visits occurring with primary care physicians (60). At 9–12 months prior to diagnosis, utilization of healthcare resources was almost 2-fold increased. “*Fever, unspecified*” and “*chest pain, unspecified*” symptoms were associated with shorter time to diagnosis. Notably, many young individuals with SLE carry psychiatric diagnoses prior to being diagnosed with SLE, which was also associated with increased pre-diagnosis healthcare use (60). Conclusively, a high index of suspicion of lupus is very important and in real-world primary care practice, SLE should be suspected in any patient who presents with unexplained manifestations involving two or more systems (61).

## Diagnostic steps in patients presenting with features suggestive of SLE: which serological labs are needed at primary care level?

Lupus is the disease with the greater number of detectable antibodies [more than 100 (62)] even though only the well-known anti-nuclear antibodies (ANA) are frequently used as screening test due to their high sensitivity (63–65). A systematic literature review and meta-regression confirmed the very high sensitivity of ANA for SLE (66). Accordingly, ANA at a titer 1:80 (by indirect immunofluorescence) has been introduced as an entry criterion for the new, under development SLE classification criteria (66). Nevertheless, 27% of a panel of international lupus experts felt comfortable to diagnose SLE even in the absence of positive ANA (67). Remarkably, ANA have been shown to predate the clinical onset of lupus and could be useful in primary care for the early recognition of the disease (68).

However, a major drawback of ANA is the low diagnostic specificity since positive test can be found in numerous other autoimmune diseases (autoimmune thyroiditis, autoimmune liver diseases and myasthenia among others) but also in healthy individuals especially at low titres (69–71). Unfortunately, in primary care, ANA is often overused as a screening test for various rheumatic illnesses including lupus. This is also the case in pediatric cases where it should not be ordered as a screening test for non-specific complaints such as musculoskeletal pain. In practical terms, ANA should be ordered only in cases of adults or children with signs and/or symptoms suggestive of SLE. By limiting ANA testing, GPs can avoid unnecessary referrals, reduce medical expenses and anxiety for the patient and her/his family (72). If ANA is negative, it could be repeated at subsequent time point only when there are new or worsening signs and symptoms pointing toward the diagnosis of SLE (73). Other specific autoantibodies are used to confirm SLE diagnosis. Specifically, anti-double strand DNA can be used for both diagnosis and evaluation of disease activity, and anti-Sm antibodies are highly specific for SLE (74).

## Diagnosis vs. classification of SLE

Diagnosis of SLE is still based on the strong clinical acumen of expert rheumatologists, since no diagnostic criteria have been validated so far (75). Both the 1997 ACR and the 2012 SLICC (58) classification criteria sets reflect the above-described picture of a multi-faceted disease, and although they have been established for epidemiological studies, they are often used in clinical routine to support the initial diagnostic thoughts (63, 76).

Relevant for the primary care setting is the observation that the SLICC 2012 criteria display overall higher sensitivity as compared with the ACR 1997 criteria, especially early in the disease (i.e., first 5 years: 89.3 vs. 76.0%, respectively) (77). Notably, in about 22% of community-based lupus cases, clinical diagnosis preceded the ACR-based definition by at least 1 year. These data suggest that, at the population level, not all individuals diagnosed with SLE by an expert rheumatologist fulfill the classification criteria (20). To this end, a Steering Committee appointed jointly by the European League Against Rheumatism (EULAR) and the ACR have been working on a new set of SLE classification criteria, developing a weighted scoring system which possibly will be helpful for the early phases of the disease, at which stage the existing criteria underperform compared to clinical SLE diagnosis (63). Although presence of more than 3 criteria makes the diagnosis of SLE probable, the opposite may not be true (75). To this end, diagnostic criteria remain an unmet need for SLE which is difficult to attain: nearly 100% sensitivity and 100% specificity would be needed and they should apply universally, therefore reliable biomarkers are needed. As Larosa et al. report in an interesting review regarding the advances in classification and diagnostic criteria, it may be over-simplistic to dichotomize as “*present*” or “*absent*” a disease that may evolve (78, 79). Nonetheless, the practical message for the GP is that the classification criteria are useful as a reminder and guide for considering the diagnosis of SLE and having any possible cases referred to specialists for further assessment.

## The concept of preclinical lupus

Current paradigm supports the notion that there is a progression from a phase of asymptomatic autoimmunity (“preclinical lupus”) through initial symptoms of the disease (incomplete lupus erythematosus) to full-spectrum SLE (complete lupus erythematosus) which can be classified also with the classification criteria. This entity of “pre-lupus” would be of value to be recognized from a primary care physician as nearly 20% of such cases will progress into full-blown SLE (80). Moreover, the term “undifferentiated connective tissue disease” (UCTD) has been used to describe the existence of signs and symptoms consistent with SLE or other defined systemic autoimmune connective tissue disease but that do not yet fulfill the classification criteria (81). At present, we cannot accurately predict which patients will eventually develop the disease (82). Incomplete lupus erythematosus presents with fewer clinical manifestations than SLE, however patients can also accrue organ damage and early mortality (83). Common features include polyarthritis or hematological disorders and on average, patients are older than those with SLE and less likely to have ANA titers ≥1:80 (83, 84).

## Lupus mimickers and overlap syndromes

Differential diagnosis is important in lupus diagnostics since many other autoimmune diseases present clinical similarities (including ANA positive tests) such as autoimmune hepatitis, dermatomyositis, inflammatory myopathies, juvenile idiopathic arthritis, primary biliary cirrhosis, rheumatoid arthritis, Sjögren's syndrome and systemic sclerosis (85). Lupus mimickers refer to a group of conditions that exhibit clinical and laboratory features resembling SLE and include infections, neoplasms and medications (85). The most frequent entity reported as a lupus mimicker is viral infection especially from parvo virus. Moreover, there are a number of overlap syndromes that combine SLE characteristics with features from other diseases including rheumatoid arthritis (also referred to as “rhupus”), polymyositis/dermatomyositis, systemic sclerosis and Sjögren's syndrome (86). In addition, the term mixed connective tissue disease (MCTD) is used to define a combination of clinical manifestations of SLE, cutaneous systemic sclerosis and polymyositis/dermatomyositis, in the presence of anti-U1-RNP antibodies. The main symptoms of this disorder include polyarthritis, hand edema, Raynaud's phenomenon, sclerodactyly, myositis and esophageal hypomobility, and studies have suggested a low frequency of evolution into another definite connective tissue disease (87).

## Drug-induced lupus and other lupus-like conditions

Drug-induced lupus refers to an autoimmune disorder that resembles SLE and is actually an idiosyncratic adverse effect of certain medications, particularly hydralazine, procainamide, isoniazid, minocycline, diltiazem and TNF inhibitors (88). Most cases appear after medium-to-long term exposure to the offending agent and tend to manifest arthralgias or arthritis, myalgia, fatigue, and serositis. The distinction from SLE is based on the history of drug exposure and the absence of specific lupus features and autoantibodies such as anti-dsDNA and Extractable Nuclear Antigen Antibodies (ENA) (89). Other organ-limited autoimmune diseases, especially autoimmune thyroid disease (90), as well as primary immunodeficiency syndromes (91), can present with mild lupus-like manifestations from the skin and joints.

## Does earlier diagnosis of SLE matter?

Population-based screening for SLE is currently not advised. Although increasing awareness of SLE has reduced diagnostic delay, still the average time from symptom onset to diagnosis is approximately 2 years (92). Probably due to the lower suspicion, a longer time lag has been reported for children, males and late-onset disease (42). Patients with less than 6 months' delay may experience lower flare rates, less healthcare utilization and costs, as compared with those with at least 6 months' delay (93). Notably, for patients with major organ disease (nephritis, neurological), delay in prompt diagnosis and initiation of immunosuppressive therapy has been linked to adverse outcomes (94–96). Additionally, failure to achieve low disease activity in the first 6 months after diagnosis has been associated with early damage accrual (95, 97). Finally, in patients with early disease, all subscales of quality of life can be improved with proper therapy over a period of 2 years (98).

## When to refer a patient with possible lupus

If the history and physical examination are not suggestive of clinically-overt disease and laboratory tests show only an isolated, low-titer positive ANA, the patient can be followed at primary care after education about warning signs and symptoms. At this point, patients warranting specialist follow-up include individuals with a family history of lupus or from high-risk ethnic descent. Co-existence of positive ANA and one or more other possible SLE features is the most frequent cause for rheumatology consultation in order to rule out SLE (75). A recent review showed that a reliable and validated tool to rate the urgency of referrals from a primary care doctor to a rheumatologist is not currently available (99).

## What are the concerns/questions of people who have lupus?

The principal concerns of lupus patients include fear of worsening so that they become dependent on others, of not being able to take care of their children, and of the possibility of transmitting SLE to their children (100). Non-adherence to recommended therapy is more common at the first stages because of the difficulty patients face in accepting a chronic disease that requires lifelong treatment (100). Interestingly, of the 10 top patient concerns, only two were common to the top 10 physician concerns and vice versa: most of what physicians rated higher, were rated lower by patients, suggesting the existence of communication gap (101). Another explanation could be the different perspectives and priorities of patients with SLE, especially the younger ones (102), such as the limitation of their capacities at physical and social level or achieving their personal goals. In this context, a GP may help to alleviate patients' concerns and improve their adherence to therapy (103).

## Severe forms of SLE

Lupus nephritis represents one of the severe complications of SLE, accounting for increased morbidity (including end-stage renal disease) and mortality (59, 104, 105), therefore early suspicion at the primary care level is of paramount importance. Neuropsychiatric lupus is also an emerging severe lupus phenotype (20) and encompasses a wide range of neurologic and psychiatric manifestations of varying severity such as seizures, cognitive dysfunction, psychosis and depression (106, 107). Of note, seizure disorder is not rare and can sometimes be the presenting manifestation of SLE. Therefore, GPs should have a low threshold to evaluate and/or refer such cases for possible SLE, as this can result in prompt initiation of immunosuppressive treatment apart from anti-epileptic intervention (108).

## Antiphospholipid syndrome (APS)

APS is the association of thrombosis and/or pregnancy morbidity with antiphospholipid antibodies (aPL) (lupus anticoagulant [LA], anticardiolipin antibodies [aCL], and/or anti-β_2_-glycoprotein-I antibodies [aβ_2_GPI]) and can occur as secondary disease in 15–20% of SLE patients (109). SLE patients with aPL have a higher prevalence of thrombosis, pregnancy morbidity, valve disease, thrombocytopenia, hemolytic anemia, renal lesions, and cognitive impairment; and higher tissue and organ damage (109, 110). At the primary care level, consecutive miscarriages and/or unexplained thrombotic event, especially in the absence of traditional risk factors, should raise the suspicion of APS (111).

## Pregnancy in lupus

As SLE is frequently diagnosed during the childbearing years, reproductive health issues are relevant to everyday practice (112). Although fertility is generally preserved and the rate of live births ranges 85–90%, still pregnancy is considered a high-risk situation for female SLE patients (113). Specifically, there is increased risk for disease flares and pregnancy-related complications such as preeclampsia (113). There is also increased risk for fetal morbidity, particularly preterm birth (relative risk [RR] 2.05) (114)], intrauterine growth restriction, and neonatal lupus. To this end, the EULAR has issued specific recommendations for the risk stratification and management of pregnancy in women with SLE (115). In this context, GPs may play an important role, especially at the pre-conception stage by assessing for exposure to any potentially harmful medications.

## SLE multi-morbidity

SLE is frequently burdened with a variety of comorbidities [cardiovascular disease, metabolic syndrome (116), malignancies (117–121), infections (122) and osteoporosis, among others] (1, 123–125). Comorbidities have an adverse impact on health-related quality of life (126), work productivity (127) and survival (128, 129) and lead to more complex management, increased hospitalizations and healthcare costs (130). Male patients have higher rates of cardiovascular disease/stroke and cancer. Conversely, female patients develop higher rates of infections and osteoporosis. Notably, patients at younger age groups are at the greatest risk for comorbid conditions compared with their healthy counterparts (123). Comorbidities can change over time in relation to patient age and use of medications, therefore their presence, severity and impact should be updated at regular intervals (131).

A recent meta-analysis revealed at least 2-fold increased *cardiovascular risk* for SLE patients, with elderly patients having the highest absolute risk and young women presenting very high relative risks as compared with the general population (132). Traditional risk factors do not fully explain the increased risk and autoimmunity (anti-phospholipid antibodies, disease activity, and inflammation) has been implicated (124). To this end, SLE has been considered an independent risk factor for ischemic heart disease (133).

In terms of *metabolic risk factors*, hypertension may be as prevalent as 75% in various SLE cohorts (124). The prevalence of dyslipidemia ranges from 36% at diagnosis to 60% after 3 years. Numerous mechanisms are implicated in its pathogenesis, including antibodies against lipoprotein lipase as well as cytokines that affect the balance and content of lipoproteins (134). Prevalence of diabetes ranges from 2.7 to 7% and increases over time after diagnosis reaching up to 14% (135). Obesity (defined as body mass index [BMI] >30 kg/m^2^] is present in about one third of patients (136).

SLE patients have nearly 2-fold increased frequency of *atopic dermatitis* (6.8 vs. 3.1%) and *asthma* (10.6 vs. 7.6%) as compared to controls (137). Further, the overall incidence rate of c*hronic obstructive pulmonary disease* was 1.7-fold higher in SLE patients than controls (138).

*Infections* are an emerging problem for SLE patients. It is estimated that 14–52% of SLE hospitalizations are due to infections including pneumonia and opportunistic infections (i.e., pneumocystis pneumonia, herpes zoster, cytomegalovirus) (139).

Finally, the pooled risk ratio (RR) for all types of *malignant disorders* in patients with SLE has been estimated to 1.28 (117–121). This risk is related to the pathology of the underlying rheumatic disease including the inflammatory process, immunological abnormalities, and exposures such as smoking and viral infections (140). In particular, SLE has been associated with increased risk of non-Hodgkin lymphoma (OR 3.02), Hodgkin lymphoma (OR 2.43) and multiple myeloma (OR 2.57) (118). SLE also is a risk factor for cervical neoplasia (119). In contrast, there is a decreased risk of hormone-sensitive cancers such as breast and prostate (141).

*Fibromyalgia* is another frequent comorbidity (range 6–22% of lupus patients) especially after the first 5 year since diagnosis. Its recognition is important for the optimal management of the disease (142, 143).

Regarding mental comorbidities, a meta-analysis revealed that the prevalence estimates of *depression* and anxiety were 30–39 and 40%, respectively in SLE patients (144) which were higher than the general population and higher than other rheumatic diseases (144). The EULAR has issued recommendations for monitoring and treating comorbidities in SLE (131).

## Preventative measures in patients with SLE

The EULAR has highlighted the benefit of smoking cessation, weight control and physical exercise as adjuvant therapy in patients with SLE, especially those with increased cardiovascular risk (145).

### Smoking cessation

Smoking has been long implicated as a trigger factor for SLE onset, characteristically, described as “*the fire behind the disease*” (146–150). Strong associations have been recently supported between current smoking and more than 10 pack-years of smoking with SLE risk (hazard ratio [HR] 1.86) and positive anti-dsDNA autoantibodies (151). Notably, smoking cessation was shown to reduce such risk to that of non-smokers suggesting that this is a modifiable risk factor (151, 152). The impact of smoking on the course of SLE is not consistent across studies, however most findings suggest increased cutaneous manifestations, flares and organ damage scores (153) as well as worse therapeutic results (154) in smokers than in non-smokers.

### Weight control

Obesity may lead to decreased functional capacity, more fatigue, and increased risk of metabolic syndrome in lupus patients. Therefore, patients should be advised to lose excessive weight (155, 156). It is important to highlight that the negative impact of obesity occurs at a lower BMI than is often considered clinically as a problem, so addressing this preventable risk factor may improve long-term outcomes (157).

### Physical activity and healthy lifestyle

Given the increased cardiovascular risk in SLE, effective interventions are suggested to improve physical activity levels (158). Barriers such as joint pain, osteoporosis, neuropathy encourage sedentary lifestyle, a physical state that is not routinely addressed by physicians during follow up. In lupus patients, enhanced quality of life and better metabolic profile were reported in more physically active patients (155). Physical activity is safe in most autoimmune diseases including SLE. Aerobic exercise has been shown to increase exercise tolerance and improve baseline oxygen consumption. A diet rich in polyunsaturated fatty acids, avoiding a sedentary lifestyle and engaging exercise under supervision may be recommended for patients with stable SLE (153).

### Cancer screening

Due to the lack of studies comparing enhanced cancer screening in lupus patients, it is currently recommended that patients with SLE should adhere to general population guidelines, with potentially enhanced screening for cervical dysplasia/cancer, especially in those who have received high-potency immunosuppressive therapy such as cyclophosphamide.

### Immunizations

Immunizations against pneumococus and influenza are recommended in SLE patients (159, 160). Live virus vaccines are contraindicated when patients are receiving immunosuppressive treatments (including prednisolone at a dose >20 mg/day), however, live attenuated vaccinations are permitted on a case-to-case basis. Vaccinations should generally be administered while the disease is under control (161). Patients with lupus who are as young as age 40 years could potentially benefit from herpes zoster vaccines (162). Moreover, available data suggest that HPV vaccines can be given safely, given the increased incidence of cervical abnormalities due to HPV in SLE, this vaccination should be offered (163).

### Preventing treatment–associated comorbidities

Glucocorticoids and antimalarial drugs are the mainstream in lupus management along with immunosuppressive or biologic drugs. Glucocorticoids are linked to adverse long-term consequences including organ damage accrual. Consequently, reduction and, when possible, complete withdrawal of glucocorticoids should be attempted (164). Emerging evidence suggests that, in chronic maintenance therapy of SLE, steroid dose should not exceed 5 mg/day of prednisone equivalent.

On the contrary, data underscore favorable effects of prolonged treatment with antimalarials, not only on controlling disease activity but also on reducing damage accrual and mortality rates. Accordingly, current recommendations suggest that antimalarial drugs such as hydroxychloroquine should be considered in all SLE patients (165, 166). Hydroxychloroquine is generally safe and may be prescribed even during pregnancy and lactation. Caution is required for the early detection of retinopathy, a rare but serious complication of the prolonged use (167). Belimumab is the first biologic approved for the treatment of lupus inhibiting B-cell activity (168) and treated patients demonstrated significant clinical improvement with a concomitant reduction or discontinuation of glucocorticoids in 70.5% (169).

*Adjunctive therapies* should be considered to control comorbidity in lupus (166), a recommendation reflecting drugs such as anti-hypertensives, lipid-lowering agents, hypoglycemics, antiplatelet/anticoagulants, and bone-protecting agents (170). These treatments are safe and efficacious in SLE patients as in the general population, although there are very few controlled studies to support benefit on long-term outcomes (171). Notably, some of the aforementioned therapies may exhibit additional benefits in patients with SLE such as the anti-proteinuric effects of renin-angiotensin axis inhibitors in lupus nephritis (172) and the use of antiplatelet agents for primary thromboprophylaxis in patients with positive antiphospholipid antibodies (173). Finally, there is weak evidence to support the use of complementary medicines in the management of the disease (170).

## The impact a GP could have on lupus care: barriers and expectations

The challenge of a model of integrated lupus care should undoubtedly point toward optimization of patient consultation. In real-world terms, this is far from ideal since many lupus patients do not routinely consult a GP and importantly, many GPs do not feel comfortable to manage such a complex disease as lupus (7). Interestingly, GPs consider SLE to be a much more severe condition than rheumatologists (and patients) actually do (8). In an older study, GPs diagnosed correctly only 11% of SLE cases presented in written scenarios, much less than rheumatologists did (174). Urowitz et al. suggested that guidelines for patients with inactive disease should be modified to include at least co-management by the rheumatologist specialized physician (175).

Lack of knowledge or education of GPs at rheumatology departments during their training may contribute to this situation. GPs usually rely on textbooks or academic tertiary care studies which emphasize on severe SLE forms with scarce reliable evidence derived from their own setting. Accordingly, it would be helpful if education efforts had a more direct relevance to GP workforce to guide their clinical practice (8, 21). Although there have been efforts to establish screening strategies to identify undiagnosed cases of SLE in the community (29, 30, 176), further studies are generally required. As SLE patients consult their general practitioner more frequently with relevant clinical features during the 5-year period prior to diagnosis, opportunities emerge to reduce diagnostic delay in primary care (176).

“Every lupus patient should have a primary care physician, who should be in regular communication with the rheumatologist and vice versa” highlights Wallace (7) and nowadays, there is substantial progress in understanding and management of lupus patients from primary care doctors (177).

Due to the multi-organ nature of the disease, patients often have to face a fragmented care system as they need even more specialized care, especially in case of severe manifestations such as renal or neuropsychiatric disease. This situation, apart from causing patient discomfort, has been associated with negative outcomes (6). Unfortunately, there is a lack of high quality evidence for interdisciplinary specialty care in the management of lupus (178).

## Conclusion

SLE is a systemic autoimmune disease that affects multiple organs. Inevitably, specialists from many disciplines often involved in the care of these patients, which however, can result in fragmented care. GPs need to deliver evidence-informed, patient-centered care, while at the same time recognizing the fact that they are restricted by their training. To overcome uncertainties and difficulties in the clinical management of SLE patients, collaboration between specialists from different disciplines and different levels of care (primary, secondary, tertiary) is of paramount importance. Developing robust evidence, tools to support informed patient decisions, and multidisciplinary shared-care pathways may facilitate this process. In this regard, the role of GPs is critical in recognizing both milder and severe presentations of SLE, navigating patients and ameliorating the disease burden at the community level.

## Author contributions

IG conceived and drafted the paper. GB supervised the work and edited the paper.

### Conflict of interest statement

The authors declare that the research was conducted in the absence of any commercial or financial relationships that could be construed as a potential conflict of interest.
